# Integrating iHEC into standardized residency training for chronic disease management: a structural equation analysis of competency outcomes via digital health platforms

**DOI:** 10.3389/fpubh.2026.1926948

**Published:** 2026-09-17

**Authors:** Dili Xie, Xuechun Sun, Jianshu Guo, Peng Liu, Junling Yang, Ping Zhong

**Affiliations:** 1 Department of Heart, Lung and Vessels, Sichuan Provincial People’s Hospital, School of Medicine, University of Electronic Science and Technology of China, Chengdu, China; 2 Department of Geriatric Cardiology, Sichuan Provincial People’s Hospital, School of Medicine, University of Electronic Science and Technology of China, Chengdu, China; 3 Department of Medical Library, Sichuan Provincial People’s Hospital, School of Medicine, University of Electronic Science and Technology of China, Chengdu, China

**Keywords:** hypertension, Intelligent Hypertension Excellence Centers, objective structured clinical examination, retrospective cohort study, standardized training of resident physicians, teaching mode

## Abstract

**Objective:**

To evaluate the association between an Intelligent Hypertension Excellence Centers (iHEC)-integrated teaching model and multidimensional educational outcomes in standardized residency training, and to explore whether self-directed learning (SDL) mediates the association between digital health-enhanced training and educational performance.

**Methods:**

A retrospective sequential cohort study was conducted among 245 resident physicians who completed a standardized 4-week hypertension rotation in the Department of Geriatric Cardiology between September 2022 and August 2024. Residents were assigned to a conventional ward-based teaching cohort (*n* = 118) or an iHEC-integrated teaching cohort (*n* = 127) according to rotation period. Both cohorts received identical curriculum requirements, faculty supervision, and assessment procedures. Educational outcomes included objective structured clinical examination (OSCE) performance, knowledge acquisition, SDL readiness, critical thinking disposition, and educational satisfaction. Structural equation modeling (SEM) with bias-corrected bootstrapping was performed to evaluate direct and indirect associations.

**Results:**

Baseline characteristics and educational assessments were comparable between cohorts. After adjustment for baseline performance, specialty, and calendar time, the iHEC cohort demonstrated higher post-rotation OSCE scores [adjusted mean difference (AMD) 6.85, 95% CI 5.12–8.58, *p* < 0.001], knowledge scores (AMD 5.42, 95% CI 3.84–7.00, *p* < 0.001), SDL scores (AMD 9.86, 95% CI 7.21–12.51, *p* < 0.001), and critical thinking scores (AMD 12.45, 95% CI 9.15–15.75, *p* < 0.001). The respecified SEM demonstrated good model fit (CFI = 0.962, TLI = 0.951, RMSEA = 0.052, SRMR = 0.038). The iHEC teaching model was positively associated with SDL (*β* = 0.68, *p* < 0.001) and educational performance (direct effect *β* = 0.36, *p* < 0.001). SDL showed a significant indirect association with educational performance (*β* = 0.38, 95% bootstrap CI 0.21–0.55), accounting for 51.4% of the total association.

**Conclusion:**

The iHEC hypertension teaching model was associated with improved resident educational outcomes, with enhanced self-directed learning representing a potential explanatory pathway underlying these observed associations; prospective multicenter studies are needed to further evaluate causal relationships, long-term retention, and implementation feasibility.

## 1 Introduction

The “Healthy China 2030” initiative has prioritized chronic disease prevention and control as a national health strategy (1). Since the nationwide implementation of standardized residency training in 2014, over 50,000 residents have enrolled annually across thousands of programs nationwide (2, 3). However, hypertension control rates in China remain below 20%, far from the World Health Organization’s (WHO) 50% target, indicating that traditional ward-based teaching models are insufficient for cultivating the longitudinal, data-driven decision-making skills essential for chronic disease management (4, 5). This workforce imperative demands pedagogical innovations that transcend episodic inpatient instruction (6).

Hypertension exemplifies this educational gap (6). As the most prevalent modifiable cardiovascular risk factor, hypertension affects approximately 270 million Chinese adults, yet only 57% are aware of their condition, 43% receive treatment, and merely 18% achieve adequate blood pressure control (4). Globally, over 1.4 billion adults live with hypertension, underscoring the universal need for competent hypertension management across all levels of healthcare (4, 5). For resident physicians rotating through geriatric cardiology—where multimorbidity and individualized blood pressure targets are paramount—the ability to manage hypertension across inpatient, outpatient, and home settings is essential (6). Conventional teaching, however, often confines hypertension education to episodic inpatient encounters, limiting residents’ exposure to the dynamic, data-rich decision-making that characterizes real-world chronic disease care (7). With only 18% of Chinese hypertensive patients achieving blood pressure control, the gap between current training outputs and population health needs represents a critical workforce capacity bottleneck in achieving national health targets (4, 5).

In response to these pedagogical limitations, digital health technologies have emerged as promising adjuncts to competency-based residency training (8). A growing body of evidence demonstrates that digital education interventions can effectively improve health professionals’ knowledge, skills, and satisfaction, performing at least as well as traditional learning methods (9, 10). Concurrently, the concept of “digital health competencies”—the specific capabilities required for healthcare professionals to effectively utilize digital technologies—has gained traction as an essential component of modern medical curricula (8, 11). A recent international consensus statement identified four core domains of digital health competencies: professionalism in digital health, patient and population digital health, health information systems, and health data science (8).

The Intelligent Hypertension Excellence Centers (iHEC) platform represents a distinctive integration of clinical management infrastructure and educational potential (12). Launched by the China Hypertension Alliance and led by the Shanghai Institute of Hypertension, iHEC utilizes Bluetooth, wireless remote networking, and wide-area network technologies to create a standardized, intelligent platform for hypertension diagnosis and management (12, 13). Beyond its clinical functions—including remote blood pressure monitoring, lifestyle tracking, follow-up alerts, and clinician feedback—the platform serves as an interactive learning environment where residents can observe longitudinal patient trajectories, connect guideline recommendations with actual clinical outcomes, and participate in supervised remote management tasks (14). Importantly, the iHEC platform operationalizes core digital health competencies—namely, interpreting real-world dynamic data, engaging in telemonitoring, and integrating digital feedback into clinical reasoning (8, 11). Lawrence et al. (15) proposed a “digital determinants of health” (DDoH) framework for understanding the intersections of health outcomes, technology, and training, arguing that this framework is critical to developing comprehensive digital health competencies in medical education. By embedding residents in a digitally enriched clinical workflow, iHEC-based teaching extends beyond static hypertension knowledge to cultivate broader adaptive expertise aligned with modern digital care delivery paradigms (11).

To date, however, the educational impact of the iHEC platform on resident training has not been systematically evaluated, and the mechanistic pathways through which digital health platforms influence multi-domain educational outcomes remain insufficiently explored (14). The present study addresses these gaps through three distinct contributions: first, it provides an empirical assessment of iHEC as an interactive teaching environment rather than merely a clinical management tool; second, it employs structural equation modeling (SEM) to evaluate an exploratory mediation mechanism—specifically, whether self-directed learning (SDL) serves as an independent pathway transmitting the association between the iHEC teaching model and overall educational performance (comprising clinical competence and critical thinking disposition)—moving beyond simple outcome comparisons to process understanding; and third, it situates this investigation within the DDoH framework and the chronic disease workforce development imperative of Healthy China 2030. We hypothesized that exposure to the iHEC-integrated model would be associated with superior educational outcomes across multiple domains—including clinical competence and critical thinking disposition—and that self-directed learning would represent a potential explanatory pathway contributing to these observed associations (16, 17).

## 2 Subjects and methods

### 2.1 Study design and cohort allocation

This retrospective, sequential cohort study was approved by the Institutional Review Board of Sichuan Provincial People’s Hospital [Approval No. 230 (2022), dated October 15, 2022]. The study was conducted and reported in accordance with the Strengthening the Reporting of Observational Studies in Epidemiology (STROBE) statement and the Template for Intervention Description and Replication (TIDieR) checklist.

The study population comprised resident physicians undergoing Standardized Training of Resident Physicians (STRP) who completed a mandatory 1-month hypertension rotation in the Department of Geriatric Cardiology between September 2022 and August 2024. Residents were allocated sequentially into two consecutive academic cohorts based on their rotation training period:

- Conventional teaching cohort: residents rotating from September 1, 2022, to August 31, 2023, received traditional ward-based clinical education.
- iHEC-integrated teaching cohort: residents rotating from September 1, 2023, to August 31, 2024, received the digital platform-assisted education.

To minimize potential confounding and avoid cross-cohort contamination, both cohorts received standardized educational training under identical core conditions, including a fixed rotation duration of 4 weeks per rotation block, the same curriculum requirements and learning objectives, the same instructional faculty and clinical preceptors, and identical pre- and post-rotation assessment procedures, scoring criteria, and assessment personnel. This design ensured that differences between cohorts were primarily attributable to the educational intervention rather than variations in training environments or evaluation processes.

Contamination was avoided because the iHEC platform was formally launched in the department on September 1, 2023; prior to this date, residents had no access to the platform or its digital tools.

All procedures complied with the Declaration of Helsinki. Given the retrospective analysis of de-identified archived educational records routinely collected during residency, the Institutional Review Board granted a waiver of written informed consent.

### 2.2 Educational interventions

#### 2.2.1 Conventional teaching model

Residents in the conventional cohort underwent traditional clinical training, consisting of bedside teaching rounds (2 h/week), didactic lectures on clinical practice guidelines (2 h/week), attending-led case discussions (1 h/week), and supervised direct outpatient/inpatient care. Faculty feedback was provided verbally during daily clinical workflow and case presentations.

#### 2.2.2 iHEC-integrated teaching model

In addition to the core traditional curriculum, residents in the iHEC cohort utilized the iHEC digital platform during their 4-week rotation:

- Platform tasks and activity: residents were required to manage real-time digitized patient profiles, perform dynamic BP trajectory monitoring, utilize embedded clinical decision-support systems (CDSS) for risk stratification, and construct standardized chronic disease management plans.
- Exposure volume: each resident was required to review and manage a minimum of 15 clinically authentic digital hypertension cases on the platform during the rotation (average completed:18.4 ± 2.6 cases).
- Supervision and feedback: attending physicians reviewed resident digital management entries bi-weekly and provided structured online feedback via the platform’s supervisor portal, supplemented by a 30-min weekly structured debrief.
- Faculty preparation and fidelity: all participating instructors underwent a mandatory 4-h orientation on platform functionality and debriefing rubrics prior to implementation. Platform usage logs were audited weekly by the departmental educational committee to ensure intervention fidelity.

### 2.3 Outcome measures and assessment procedures

The prespecified primary outcome was post-rotation clinical competence measured by OSCE score. Secondary outcomes included knowledge acquisition, self-directed learning readiness, critical thinking disposition, and educational satisfaction. All outcomes were assessed at baseline (Day 1) and after completion of the 4-week rotation (Day 28).

#### 2.3.1 Clinical competence (objective structured clinical examination, OSCE)

Clinical performance was evaluated using a standardized 4-station hypertension-specific OSCE:

Station 1: cardiovascular history taking and risk factor assessment;

Station 2: physical examination and accurate BP measurement;

Station 3: diagnostic reasoning and electrocardiogram/lab interpretation;

Station 4: patient counseling and antihypertensive pharmacotherapy management.

Each station lasted 10 min and was evaluated using a checklist rubric (0–100 points per station, overall OSCE score averaged across stations). Assessors were clinical faculty blinded to the residents’ cohort assignment. Inter-rater reliability was high, with an intraclass correlation coefficient (ICC) of 0.88 (95%CI, 0.83 ~ 0.92) established across evaluation sessions.

#### 2.3.2 Knowledge acquisition assessment

Knowledge was evaluated via a 50-item multiple-choice examination covering clinical hypertension guidelines, secondary hypertension workup, and hypertensive emergencies (score range: 0–100). Parallel test forms with proven item equivalence were used for pre- and post-rotation testing. Cronbach’s\alpha in the present sample was 0.84.

#### 2.3.3 Self-directed learning readiness scale for medical students (SRSSDL)

Self-directed learning readiness was assessed using the SDLRS, originally developed by Fisher et al. (18) and subsequently validated in medical education settings (19). The scale evaluates three dimensions: self-management, desire for learning, and self-control. The instrument Cronbach’sα for the total scale in this sample was 0.91 (subdomains ranged from 0.81 to 0.87). To prevent mathematical redundancy in latent variable modeling, individual subdomain scores were utilized as primary indicators, and the scale total score was excluded from the measurement model.

#### 2.3.4 California critical thinking disposition inventory (CCTDI—Chinese version)

Critical thinking disposition was measured using the validated Chinese adaptation of the CCTDI (20). The inventory utilizes 6 established dimensions relevant to Chinese healthcare trainees: Open-Mindedness, Analyticity, Systematicity, Critical Thinking Self-Confidence, Inquisitiveness, and Cognitive Maturity (Truth-Seeking was omitted in accordance with the validated Chinese structural revision). Items were scored on a 6-point Likert scale (subscale scores: 10–60). In the present study, overall Cronbach’s *α* was 0.89.

#### 2.3.5 Educational satisfaction

Overall rotation satisfaction was evaluated post-rotation using a 5-item Likert questionnaire (1 = Very Dissatisfied, 5 = Very Satisfied; range 5–25), covering teaching quality, learning environment, supervision adequacy, and digital tool utility.

### 2.4 Structural equation modeling (SEM) framework

To address conceptual circularity and establish a coherent, identified model, the analytic framework explicitly separated self-directed learning (SDL) from the outcome construct. SDL was specified purely as an independent mediator transmitting the indirect association between teaching model and overall educational performance.

#### 2.4.1 Measurement model

Confirmatory factor analysis (CFA) was initially performed to validate the measurement structure of the latent constructs. Self-directed learning (first-order mediator) was represented by three subdomains: self-management, desire for learning, and self-control. Clinical competence (first-order outcome factor) was assessed using post-rotation OSCE and knowledge scores. Critical thinking (first-order outcome factor) was measured by the six subdomains of the CCTDI. Educational performance was modeled as a second-order latent construct reflected by the two first-order factors, namely clinical competence and critical thinking.

Construct validity was assessed using standardized factor loadings (>0.50), composite reliability (CR > 0.70), average variance extracted (AVE > 0.50), and the heterotrait–monotrait ratio (HTMT <0.85) for discriminant validity. To further evaluate the robustness of the specified measurement structure, alternative measurement models were examined, including a single-factor model and a correlated first-order factor model. These alternative specifications were compared with the hypothesized model based on overall model fit indices (*χ*^2^/df, CFI, TLI, RMSEA, and SRMR), with preference given to the model demonstrating superior fit and theoretical coherence.

#### 2.4.2 Structural model and mediation analysis

After confirming the measurement model, a SEM was constructed to evaluate direct and indirect associations among the teaching model, self-directed learning, and educational performance.

The exogenous variable was the teaching model (0 = conventional teaching, 1 = iHEC). Self-directed learning was specified as the mediator (first-order latent variable), and educational performance was specified as the endogenous outcome (second-order latent variable). The model was adjusted for potential confounders, including calendar time (training year), baseline performance scores (baseline OSCE, knowledge score, SRSSDL, and CCTDI), residency training year, and specialty.

The following structural paths were examined:

- *a path:* teaching model → self-directed learning
- *b path:* self-directed learning → educational performance
- *Direct effect (c′ path):* teaching model → educational performance
- *Total effect (c):* c = c′ + (a × b)

Given the non-randomized observational design, mediation effects were interpreted as exploratory model-based indirect associations rather than evidence of definitive causal mediation.

#### 2.4.3 Estimation and software

SEM analyses were conducted using Mplus version 8.3 (Muthén & Muthén, Los Angeles, CA, USA). Parameters were estimated using maximum likelihood estimation with robust standard errors (MLR) to account for potential deviations from normality. Because residents were trained within standardized rotation blocks, rotation block was included as a covariate in the structural equations to account for possible block-level variation. Cluster-robust estimation was not additionally applied because all rotation blocks followed the same curriculum, were supervised by the same instructional faculty, and used identical assessment procedures. Standardized path coefficients (*β*), unstandardized estimates (*B*), and standard errors (SE) were reported.

The significance of indirect effects was evaluated using bias-corrected bootstrapping with 5,000 resamples. An indirect effect was considered statistically significant when the corresponding 95% confidence interval (CI) did not include zero.

#### 2.4.4 Model fit assessment

Model fit was evaluated using commonly accepted goodness-of-fit criteria:

- *χ*^2^/df < 3.0
- Comparative fit index (CFI) > 0.95
- Tucker–Lewis index (TLI) > 0.95
- Root mean square error of approximation (RMSEA) < 0.06 (90% CI reported)
- Standardized root mean square residual (SRMR) < 0.08

### 2.5 Data management and statistical analysis

#### 2.5.1 Data cleaning and assumption testing

Data verification and descriptive analyses were performed using SPSS version 28.0 (IBM Corp., Armonk, NY, USA). Continuous variables were assessed for distributional assumptions using Q–Q plots and the Shapiro–Wilk test. Continuous indicators were standardized using *z*-score transformation before SEM analyses to facilitate comparison of standardized coefficients.

The final analytic dataset included complete records for all 245 residents across baseline and post-rotation assessments; therefore, no missing data imputation or full information maximum likelihood (FIML) procedure was required.

#### 2.5.2 Descriptive and comparative analyses

Continuous variables are presented as mean ± standard deviation (SD) for normally distributed data or median (interquartile range, IQR) for non-normally distributed or ordinal data. Categorical variables are summarized as numbers and percentages (*n*, %).

Baseline comparability and unadjusted differences in post-rotation outcomes between cohorts were assessed using independent-samples *t* tests or Mann–Whitney *U* tests, as appropriate. Within-group pre- and post-rotation changes were evaluated using paired-samples *t* tests. Categorical variables were compared using Pearson’s chi-square test or Fisher’s exact test.

Adjusted between-group differences were estimated using analysis of covariance (ANCOVA), with adjustment for baseline outcome values, specialty, and calendar period. Standardized effect sizes were calculated using Cohen’s d, with values of 0.2, 0.5, and 0.8 indicating small, moderate, and large effects, respectively.

To reduce type I error inflation from multiple secondary comparisons involving scale subdomains, the Benjamini–Hochberg false discovery rate (FDR) correction was applied, with *q* < 0.05 considered statistically significant. All statistical tests were two-sided, and *p* < 0.05 was considered statistically significant.

Sensitivity analyses were performed using sequentially adjusted models. Model 1 was the primary ANCOVA model adjusted for baseline OSCE score, specialty, and calendar time. Model 2 additionally included residency training year. Model 3 further adjusted for rotation block. Model 4 additionally incorporated baseline academic performance indicators, including baseline knowledge score, baseline SRSSDL score, and baseline CCTDI score. The robustness of the association between teaching model exposure and post-rotation educational outcomes was evaluated across these progressively adjusted models.

## 3 Results

### 3.1 Cohort flow and record completeness

A total of 262 resident physicians rotating through the Department of Geriatric Cardiology were screened for eligibility. As detailed in the STROBE flow diagram, 17 residents were excluded due to interrupted rotations (*n* = 8), missing baseline evaluation records (*n* = 5), or incomplete post-rotation outcome forms (*n* = 4).

The final analytic sample comprised 245 eligible resident physicians:

Conventional teaching cohort: *n* = 118 (rotating between September 1, 2022, and August 31, 2023).

iHEC-integrated teaching cohort: *n* = 127 (rotating between September 1, 2023, and August 31, 2024).

All 245 included participants had complete archived paired pre- and post-rotation records across all primary and secondary evaluation domains without any missing data points. Consequently, no missing data imputation was performed, and no FIML procedure was required during model estimation.

### 3.2 Baseline characteristics and educational outcomes

#### 3.2.1 Baseline comparability

As shown in Table 1, baseline demographic and educational characteristics were well balanced between the conventional (*n* = 118) and iHEC (*n* = 127) cohorts. The conventional cohort included 63 males (53.4%) and 55 females (46.6%), with a mean age of 25.14 ± 1.52 years, whereas the iHEC cohort included 70 males (55.1%) and 57 females (44.9%), with a mean age of 25.02 ± 1.63 years (*p* = 0.552).

**Table 1:** Baseline characteristics, pre- and post-rotation educational outcomes, and between-cohort comparisons (*N* = 245).

Parameter/assessment domain	Conventional cohort (*n* = 118)	iHEC cohort (*n* = 127)	Statistical test (*t*/*χ*^2^/*U*)	Unadjusted *p*-value	Adjusted between-group difference (95% CI)^a^	FDR adjusted *q*-value	Effect size (Cohen’s *d*)
Demographics							
Age (years, Mean ± SD)	25.14 ± 1.52	25.02 ± 1.63	*t* = 0.596	0.552	—	—	—
Sex [male/female, *n* (%)]	63 (53.4) / 55 (46.6)	70 (55.1)/57 (44.9)	*χ*^2^ = 0.072	0.788	—	—	—
Clinical competence (OSCE)							
Baseline	68.42 ± 6.15	68.15 ± 5.88	*t* = 0.350	0.727	—	—	—
Post-rotation	76.85 ± 5.92	83.92 ± 5.48	*t* = 9.689	<0.001	+6.85 (5.12 to 8.58)	<0.001	0.82
Change (Δ post − pre)	+8.43 ± 4.12^b^	+15.77 ± 4.85^b^	*t* = 12.751	<0.001	—	—	—
Knowledge acquisition							
Baseline	71.20 ± 6.80	70.70 ± 6.45	*t* = 0.595	0.552	—	—	—
Post-rotation	78.15 ± 6.10	83.75 ± 5.85	*t* = 7.323	<0.001	+5.42 (3.84 to 7.00)	<0.001	0.71
Change (Δ post − pre)	+6.95 ± 3.85^b^	+13.05 ± 4.22^b^	*t* = 11.802	<0.001	—	—	—
Self-directed learning (SRSSDL)							
Baseline (total score)	135.80 ± 12.40	135.10 ± 12.10	*t* = 0.440	0.661	—	—	—
Post-rotation (total score)	146.50 ± 11.80	156.80 ± 11.20	*t* = 7.009	<0.001	+9.86 (7.21 to 12.51)	<0.001	0.78
Self-management subdomain	48.10 ± 4.20	51.80 ± 3.90	*t* = 7.142	<0.001	+3.58 (2.59 to 4.57)	<0.001	0.73
Desire for learning subdomain	49.20 ± 4.10	52.90 ± 3.80	*t* = 7.329	<0.001	+3.61 (2.64 to 4.58)	<0.001	0.75
Self-control subdomain	49.20 ± 4.10	52.10 ± 4.00	*t* = 5.602	<0.001	+2.79 (1.81 to 3.77)	<0.001	0.58
Critical thinking (CCTDI)							
Baseline (total score)	238.50 ± 18.20	237.70 ± 17.50	*t* = 0.382	0.703	—	—	—
Post-rotation (total score)	252.10 ± 16.10	264.80 ± 15.20	*t* = 6.347	<0.001	+12.45 (9.15 to 15.75)	<0.001	0.75
Satisfaction score (5–25)	19.0 (17.0–21.0)	23.0 (21.0–24.0)	*U* = 3142.5	<0.001	—	<0.001	—

Continuous data presented as Mean ± SD or Median (IQR). Baseline comparison used independent-samples *t* tests or Chi-square tests.

a ANCOVA adjusting for baseline score, training year, specialty, and calendar time.

b Paired *t*-test within-group change from baseline *p* < 0.001.

FDR, Benjamini–Hochberg false discovery rate.

No significant differences were observed between cohorts in baseline educational assessments, including OSCE scores (*p* = 0.727), knowledge scores (*p* = 0.552), SRSSDL scores (*p* = 0.661), and CCTDI scores (*p* = 0.703), indicating comparable baseline characteristics before implementation of the teaching intervention.

#### 3.2.2 Primary and secondary educational outcomes

After the 4-week rotation, both cohorts demonstrated significant within-group improvements in all competency-related outcomes (all paired *t*-test *p* < 0.001; Table 1).

In both unadjusted comparisons and ANCOVA models adjusted for baseline performance, specialty, and calendar time, the iHEC cohort achieved significantly higher post-rotation outcomes across all evaluated domains:

- *OSCE score:* Adjusted mean difference, 6.85 points (95% CI, 5.12–8.58; *p* < 0.001; Cohen’s *d* = 0.82), indicating a large effect size.
- *Knowledge score:* Adjusted mean difference, 5.42 points (95% CI, 3.84–7.00; *p* < 0.001; Cohen’s *d* = 0.71), indicating a moderate-to-large effect size.
- *Self-directed learning (SRSSDL total score):* Adjusted mean difference, 9.86 points (95% CI, 7.21–12.51; *p* < 0.001; Cohen’s *d* = 0.78).
- *Critical thinking disposition (CCTDI total score):* Adjusted mean difference, 12.45 points (95% CI, 9.15–15.75; *p* < 0.001; Cohen’s *d* = 0.75).
- *Educational satisfaction:* Post-rotation satisfaction was significantly higher in the iHEC cohort than in the conventional cohort [median, 23.0 (IQR, 21.0–24.0) vs. 19.0 (IQR, 17.0–21.0); Mann–Whitney *U* = 3142.5; *p* < 0.001].

All post-rotation between-group differences remained statistically significant after correction for multiple comparisons using the Benjamini–Hochberg false discovery rate procedure (all *q* < 0.001).

Sensitivity analyses demonstrated that the association between iHEC-integrated teaching and improved OSCE performance remained stable after sequential adjustment for additional potential confounders. The adjusted mean difference remained statistically significant across all models (range, 6.48–6.85 points; all *p* < 0.001), indicating that the findings were robust to adjustment for residency training year, specialty, rotation block, and baseline academic performance (Supplementary Table 1).

Values represent adjusted mean differences in post-rotation OSCE scores between the iHEC-integrated teaching cohort and conventional teaching cohort. All models were estimated using ANCOVA. Model 1 corresponds to the prespecified primary adjusted analysis. Subsequent models sequentially incorporated additional measured confounders, including rotation block and baseline educational characteristics. The association remained statistically significant across all sensitivity analyses.

### 3.3 Measurement model validation and discriminant validity

CFA was conducted on the respecified measurement framework. In accordance with the revised specification, SRSSDL total score was excluded to prevent mathematical redundancy, and its three subdomains served as indicators for the mediator SDL.

The outcome construct educational performance was specified as a second-order factor reflected by two first-order factors: clinical competence (OSCE and knowledge) and critical thinking disposition (6 CCTDI subdomains).

#### 3.3.1 Convergent validity and reliability

As presented in Table 2, all standardized factor loadings for observed indicators onto their respective latent constructs were robust and statistically significant, ranging from 0.78 to 0.93 (all *p* < 0.001). Composite Reliability (CR) values across all latent variables exceeded the recommended threshold of 0.70 (range: 0.912 ~ 0.937), and Average Variance Extracted (AVE) values exceeded 0.50 (range: 0.712 ~ 0.857), confirming excellent internal consistency and convergent validity.

**Table 2:** Measurement model verification: Factor loadings, composite reliability (CR), and average variance extracted (AVE).

Latent construct/indicator variable	Standardized loading (*λ*)	Standard error (SE)	*p*-value	Composite reliability (CR)	Average variance extracted (AVE)
Self-directed learning (mediator, M)				0.912	0.776
SRSSDL: self-management subdomain	0.89	0.021	<0.001		
SRSSDL: desire for learning subdomain	0.88	0.022	<0.001		
SRSSDL: self-control subdomain	0.87	0.023	<0.001		
Clinical competence (first-order outcome)				0.923	0.857
Post-rotation OSCE score	0.93	0.015	<0.001		
Post-rotation knowledge score	0.92	0.016	<0.001		
Critical thinking (first-order outcome)				0.937	0.712
CCTDI: open-mindedness	0.82	0.024	<0.001		
CCTDI: analyticity	0.85	0.022	<0.001		
CCTDI: systematicity	0.86	0.021	<0.001		
CCTDI: critical thinking self-confidence	0.88	0.02	<0.001		
CCTDI: inquisitiveness	0.84	0.023	<0.001		
CCTDI: cognitive maturity	0.81	0.025	<0.001		
Educational performance (second-order)				0.895	0.81
Clinical competence	0.91	0.018	<0.001		
Critical thinking disposition	0.89	0.019	<0.001		

#### 3.3.2 Discriminant validity

Discriminant validity among the first-order latent constructs was evaluated using the heterotrait–monotrait ratio of correlations (HTMT) and the Fornell–Larcker criterion (Table 3). All HTMT values were well below the stringent cutoff of 0.85 (maximum HTMT = 0.72), and the square root of the AVE for each construct exceeded its highest correlation with any other construct, establishing clear discriminant validity between the mediator (SDL) and outcome components.

**Table 3:** Factor correlation matrix, Fornell–Larcker criterion, and HTMT ratios for discriminant validity.

Latent construct	(1) Self-directed learning	(2) Clinical competence	(3) Critical thinking
(1) Self-directed learning	0.881	0.68	0.62
(2) Clinical competence	0.59	0.926	0.74
(3) Critical thinking	0.54	0.65	0.844

Diagonal values are √AVE; below-diagonal values are factor correlations; above-diagonal italic values are HTMT ratios (<0.85).

### 3.4 Respecified SEM and mediation results

Following CFA confirmation, the structural equation model was fitted using Mplus 8.3 with maximum likelihood estimation with robust standard errors (MLR). Covariates including calendar time (training year), baseline scores, specialty, and rotation block were controlled within the structural equations.

#### 3.4.1 Direct structural paths

Structural path estimates (Figure 1, Table 4) demonstrated that exposure to the iHEC-integrated teaching model was positively associated with both SDL and educational performance.

**Figure 1:**
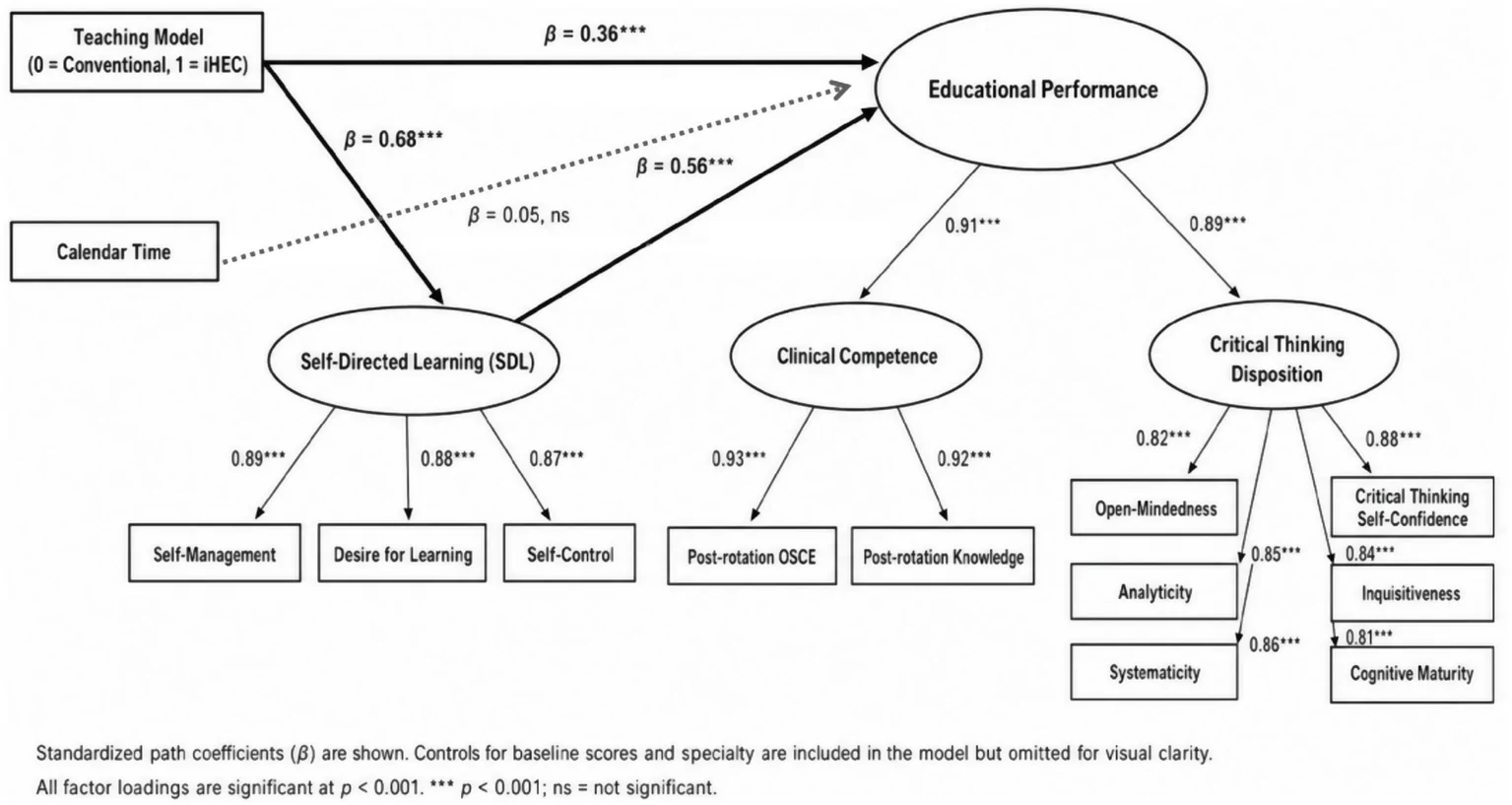
Respecified structural equation model path diagram.

**Table 4:** Structural model estimates: direct, indirect, and total effects with 5,000 bootstrap confidence intervals.

Pathway description	Unstandardized estimate (*B*)	Standard error (SE)	Standardized estimate (*β*)	*z*-value	*p*-value	95% bias-corrected bootstrap CI (lower, upper)
Direct structural paths						
a: Teaching model → SDL	6.52	0.58	0.68	11.24	<0.001	(0.56, 0.80)
b: SDL → educational performance	0.74	0.09	0.56	8.22	<0.001	(0.42, 0.70)
c′: Teaching model → performance (direct)	4.56	1.01	0.36	4.51	<0.001	(0.20, 0.52)
Covariate: calendar time → performance	0.42	0.52	0.05	0.81	0.42	(−0.03, 0.13)
Mediation analysis parameters						
Indirect path (a × b)	4.82	0.09	0.38	4.22	<0.001	(0.21, 0.55)
Total effect (c = c′ + a × b)	9.38	0.08	0.74	9.25	<0.001	(0.58, 0.90)
Proportion mediated (%)	—	—	51.40%	—	—	—

The estimated paths were as follows:

- *Path a (teaching model → SDL): β* = 0.68, SE = 0.06, *p* < 0.001.
- *Path b (SDL → educational performance): β* = 0.56, SE = 0.07, *p* < 0.001.
- *Direct effect (c′, teaching model → educational performance): β* = 0.36, SE = 0.08, *p* < 0.001.

Calendar time was not significantly associated with educational performance (*β* = 0.05, SE = 0.04, *p* = 0.420), suggesting no substantial secular trend across study years.

#### 3.4.2 Bootstrapped mediation analysis

The indirect association was assessed using 5,000 bias-corrected bootstrap resamples (Table 4).

The indirect effect of the teaching model on educational performance through SDL was statistically significant (*β* = 0.38; unstandardized *B* = 4.82; SE = 0.09; 95% bootstrap CI, 0.21–0.55; *p* < 0.001).

The total effect was also significant (*β* = 0.74; unstandardized *B* = 9.38; SE = 0.08; 95% bootstrap CI, 0.58–0.90; *p* < 0.001). The indirect pathway through SDL accounted for 51.4% of the total association.

Because the direct effect remained significant after inclusion of the mediator, SDL was identified as a partial mediator. Given the non-randomized observational design and concurrent post-rotation assessments, these mediation findings should be interpreted as exploratory statistical associations rather than evidence of causal mediation.

### 3.5 Overall model fit evaluation

The respecified measurement and structural equation models showed excellent fit to the observed data across all evaluated indices. Alternative measurement model comparisons were also performed. The hypothesized hierarchical model demonstrated better overall fit and greater theoretical consistency with the prespecified framework than simplified alternative structures, supporting retention of the final SEM specification.

- *χ*^2^ = 148.24, df = 68, *χ*^2^/df = 2.18, indicating acceptable model parsimony (<3.0);
- Comparative fit index (CFI) = 0.962;
- Tucker–Lewis index (TLI) = 0.951;
- Root mean square error of approximation (RMSEA) = 0.052 (90% CI, 0.041–0.063);
- Standardized root mean square residual (SRMR) = 0.038.

All fit indices met the prespecified criteria (CFI and TLI > 0.95, RMSEA <0.06, and SRMR <0.08), indicating that the respecified SEM provided an adequate and parsimonious representation of the relationships among teaching model exposure, self-directed learning, and educational performance.

## 4 Discussion

This retrospective sequential cohort study evaluated the integration of a digital chronic disease management platform (iHEC) into residency hypertension training. By applying a theoretically informed and non-redundant SEM, this study provides empirical support for three key educational implications.

First, a clinically authentic hypertension management platform can be repurposed as an interactive learning environment, extending the role of digital health infrastructure beyond direct patient care into graduate medical education. Second, exploratory mediation analysis suggested that SDL accounted for 51.4% of the statistical association between iHEC exposure and overall educational performance, which incorporated clinical competence and critical thinking. This finding suggests that autonomous learning activation may represent one potential pathway contributing to the association between digitally enriched learning environments and resident development. Third, by positioning this approach within the DDoH framework and chronic disease workforce development priorities (1, 4), our findings suggest that technology-enhanced education may cultivate competencies relevant to longitudinal, data-driven chronic care.

### 4.1 Underlying mechanisms and theoretical integration

Several interconnected mechanisms may explain the observed associations, with self-regulated learning (SRL) theory providing the primary conceptual framework (17). SRL involves cyclical processes of forethought, performance regulation, and self-reflection, which facilitate continuous knowledge construction and professional development (17, 21).

First, the iHEC platform may address a fundamental limitation of conventional chronic disease training: the mismatch between episodic inpatient exposure and the longitudinal nature of hypertension management (6, 22). Traditional rotation-based teaching often exposes residents to patients during acute hypertensive events or incidental admissions, resulting in fragmented understanding of disease trajectories (6). In contrast, iHEC provides continuous access to home blood pressure records, medication adherence information, lifestyle indicators, and longitudinal follow-up data (14). This repeated exposure to real-world clinical trajectories allows residents to observe how therapeutic decisions influence patient outcomes over time, thereby embedding clinical reasoning within authentic chronic care contexts (14, 23).

The adjustment for calendar time further supports the robustness of these findings. Calendar time was not significantly associated with educational performance (*β* = 0.05, *p* = 0.420), and adjustment for this factor did not materially change the estimated association between iHEC exposure and educational outcomes. However, this finding should not be interpreted as definitive evidence excluding temporal confounding, as unmeasured changes in resident characteristics, faculty experience, or institutional practices may still contribute to observed differences (24).

Second, the iHEC environment appears to promote SDL, which represents a central competency for lifelong professional development. Within the iHEC cohort, residents actively reviewed digital patient panels, developed evidence-based management strategies, consulted clinical decision support systems (CDSS), and refined their approaches based on structured feedback from supervisors (14). This iterative process of information acquisition, reflection, and adjustment closely corresponds with the principles of autonomous learning.

Importantly, SEM analysis indicated that SDL, represented by self-management, desire for learning, and self-control domains, functioned as a significant mediator, accounting for 51.4% of the total statistical association between iHEC exposure and educational performance. This suggests that the educational value of digital platforms may arise not only from providing additional clinical information but also from stimulating residents’ active engagement and autonomous learning behaviors (17, 20). The remaining proportion of the association may reflect other educational components embedded within the iHEC system, including more frequent feedback, broader case exposure, and visualization of longitudinal treatment trends, which may independently enhance pattern recognition and clinical reasoning.

Third, the iHEC model may facilitate the development of critical thinking dispositions by exposing residents to the uncertainty and complexity inherent in chronic disease management (16). Unlike many acute-care scenarios with relatively standardized pathways, hypertension management requires continuous risk–benefit assessment, individualized treatment adjustment, and consideration of patient preferences and social determinants (6, 7). Exposure to diverse and dynamic clinical trajectories may promote analytical thinking, open-mindedness, and reflective judgment, which are core components assessed by the CCTDI (16). The strong contribution of critical thinking to the second-order Educational Performance construct (*β* = 0.89) further emphasizes the importance of uncertainty management as a core competency in chronic disease care (15, 16).

### 4.2 Integration with existing literature and DDoH framework

Our findings extend previous research on digital health education in several important ways.

First, previous systematic reviews have demonstrated that digital learning approaches can achieve outcomes comparable or superior to traditional teaching methods (9, 10). However, most studies have focused primarily on effectiveness rather than underlying mechanisms. By identifying SDL as a potential mediator, this study extends the discussion from whether digital teaching works to how digitally enriched environments may facilitate learning processes (17, 20).

Second, this study provides quantitative support for the application of the DDoH framework in medical education. Lawrence et al. proposed the DDoH framework to describe the interaction between digital technologies, health outcomes, and workforce development, although empirical evidence within residency education remains limited (15). In our SEM model, clinical competence and critical thinking demonstrated strong contributions to the higher-order Educational Performance construct (*β* = 0.89–0.91), suggesting that digitally enhanced learning environments may promote coordinated improvements across multiple competency domains rather than isolated skill acquisition.

Third, the iHEC model aligns residency education with the workforce requirements of chronic disease management under the Healthy China 2030 framework (1, 4). Effective hypertension control requires healthcare professionals capable of continuous monitoring, risk assessment, and longitudinal patient management. By shifting training exposure from episodic encounters toward platform-supported population-based care, iHEC represents a potential approach for aligning medical education with evolving healthcare system needs (1, 4, 25).

### 4.3 Policy implications and systems application

At the health system level, the iHEC model represents an educational innovation that connects residency training with population health priorities (1, 4). Rather than simply transferring traditional teaching materials into digital formats, the platform restructures the learning environment around continuous clinical data, feedback loops, and longitudinal patient management.

Beyond hypertension, similar platform-supported educational approaches may be applicable to other chronic conditions requiring sustained monitoring and individualized management, including diabetes mellitus, heart failure, and chronic kidney disease (25, 26). In resource-limited primary care settings or regional training centers where experienced clinical educators are insufficient, scalable digital platforms may help expand teaching capacity and improve educational consistency (27).

Future implementation studies should investigate integration of platform-derived learning metrics into competency-based medical education frameworks, evaluate faculty workload redistribution, and assess the cost-effectiveness of digital teaching models compared with traditional faculty-intensive approaches.

### 4.4 Study limitations

Several limitations should be considered. First, the retrospective sequential cohort design limits causal interpretation. Although baseline performance and calendar time were adjusted, allocation to teaching models was not randomized. Furthermore, SDL and post-rotation outcomes were assessed concurrently, limiting definitive temporal inference regarding mediation pathways. Therefore, the mediation estimates should be interpreted as exploratory statistical associations rather than confirmatory causal mechanisms (22, 23).

Second, this study was conducted in a single geriatric cardiology department within a tertiary academic hospital. The generalizability of these findings to other specialties, primary care training environments, or institutions with different digital infrastructure requires further validation (6).

Third, educational outcomes were evaluated immediately after the 4-week rotation. Whether these competency improvements persist over longer follow-up periods or translate into improved clinical performance in independent practice remains unknown (14).

Fourth, although measured covariates were adjusted, residual confounding from unmeasured trainee characteristics, including baseline digital literacy, intrinsic motivation, or differences in faculty teaching styles, cannot be completely excluded (3, 19). Although rotation block was statistically controlled, residual clustering effects related to individual learning environments or faculty–resident interactions cannot be completely excluded.

Finally, this study did not assess implementation costs, software maintenance requirements, or potential effects on faculty workload and burnout (24, 26). Future research should incorporate these implementation outcomes to determine the feasibility and scalability of digital teaching systems.

## 5 Conclusion

This retrospective sequential cohort study suggests that an iHEC-integrated hypertension teaching model is associated with improved educational outcomes among resident physicians compared with conventional ward-based instruction. SEM analysis identified SDL as an important explanatory pathway, accounting for 51.4% of the total statistical association between iHEC exposure and educational performance.

By demonstrating the potential of repurposing a clinical management platform as a multidimensional educational environment, this study provides preliminary evidence supporting the integration of digital health infrastructure into residency training. Although prospective multicenter randomized studies are required to establish causal effects and evaluate long-term competency retention, the iHEC-integrated model represents a promising strategy for aligning graduate medical education with the increasing demands of chronic disease management in modern healthcare systems.

## Data Availability

The raw data supporting the conclusions of this article will be made available by the authors, without undue reservation.
