# Supplementary material for: Integrating iHEC into standardized residency training for chronic disease management: a structural equation analysis of competency outcomes via digital health platforms
Source: Front Public Health. 2026 Sep 17;14:1926948. doi: 10.3389/fpubh.2026.1926948 (PMC13627101; doi:10.3389/fpubh.2026.1926948)
Supplement: Supplementary file 1 [file Table_1.docx]

**Supplementary Table S1. Sensitivity analyses evaluating the robustness of the association between iHEC-integrated teaching model and post-rotation OSCE performance**

| **Model** | **Adjustment variables** | **iHEC teaching model coefficient (Adjusted mean difference, points)** | **95% CI** | **P value** | **Interpretation** |
| --- | --- | --- | --- | --- | --- |
| Model 1 (Primary model) | Baseline OSCE score, residency training year, specialty, and calendar time | 6.85 | 5.12–8.58 | <0.001 | Primary ANCOVA model |
| Model 2 | Model 1 + rotation block | 6.7 | 5.00–8.40 | <0.001 | Adjustment for potential block-level variation |
| Model 3 | Model 2 + baseline knowledge score | 6.58 | 4.88–8.28 | <0.001 | Additional adjustment for baseline cognitive performance |
| Model 4 | Model 3 + baseline SRSSDL score and baseline CCTDI score | 6.45 | 4.73–8.17 | <0.001 | Fully adjusted sensitivity model |

Values represent adjusted mean differences in post-rotation OSCE scores between the iHEC-integrated teaching cohort and conventional teaching cohort. All models were estimated using ANCOVA. Model 1 corresponds to the prespecified primary adjusted analysis. Subsequent models sequentially incorporated additional measured confounders, including rotation block and baseline educational characteristics. The association remained statistically significant across all sensitivity analyses.
